# Aerosol microphysics and chemistry reveal the COVID19 lockdown impact on urban air quality

**DOI:** 10.1038/s41598-021-93650-6

**Published:** 2021-07-14

**Authors:** Konstantinos Eleftheriadis, Maria I. Gini, Evangelia Diapouli, Stergios Vratolis, Vasiliki Vasilatou, Prodromos Fetfatzis, Manousos I. Manousakas

**Affiliations:** 1 Environmental Radioactivity Laboratory, INRASTES, NCSR Demokritos, 15310 Ag. Paraskevi, Athens, Greece; 2 LAC, Paul Scherrer Institute, Villigen PSI, Switzerland

**Keywords:** Environmental chemistry, Environmental impact, Atmospheric chemistry, Atmospheric dynamics, Climate sciences, Environmental sciences

## Abstract

Air quality in urban areas and megacities is dependent on emissions, physicochemical process and atmospheric conditions in a complex manner. The impact on air quality metrics of the COVID-19 lockdown measures was evaluated during two periods in Athens, Greece. The first period involved stoppage of educational and recreational activities and the second severe restrictions to all but necessary transport and workplace activities. Fresh traffic emissions and their aerosol products in terms of ultrafine nuclei particles and nitrates showed the most significant reduction especially during the 2nd period (40–50%). Carbonaceous aerosol both from fossil fuel emissions and biomass burning, as well as aging ultrafine and accumulation mode particles showed an increase of 10–20% of average before showing a decline (5 to 30%). It is found that removal of small nuclei and Aitken modes increased growth rates and migration of condensable species to larger particles maintaining aerosol volume.

## Introduction

A large fraction of the EU-28 urban population is still exposed to levels above the WHO (World Health Organization) guidelines (42–52% for PM10 and 74–81% for PM2.5)^1^. The development of effective control measures is challenging, while an ongoing discussion exists on the metrics best reflecting the impacts of anthropogenic sources and their health implications, for example regarding black carbon and aerosol ultrafine particles^2,3^. In March 2020, a number of mitigation measures against the spread of the COVID-19 pandemic were gradually applied in Greece. Operation of educational institutions at all levels was suspended nationwide from March 11th. Within 2 days, all commercial and social recreation activities were also suspended (close down of all cafes, bars, museums, shopping centres, sports facilities, restaurants and retail shops), except for those related to basic needs (such as food stores, pharmacies and gas stations). Finally, on March 22nd, lockdown measures were applied, with the restriction of all non-essential travel throughout the country.

The present work aims to investigate the impact of the COVID-19 control measures to key aerosol parameters and air quality metrics in the city of Athens. A number of aerosol microphysical and chemical properties, monitored at the NCSR Demokritos urban background station, are analysed, in order to understand how the changes in commercial and other activities have affected the aerosol emissions sources and the corresponding aerosol mixture within the Athens Metropolitan Area (AMA).

Athens is situated in the south part of the Greek-Balkan peninsula, relatively isolated from large urban agglomerations and surrounded by sea. Fresh air pollutants in the urban area mainly originate from the city and surrounding region itself. Even though it may often be affected by long range transport^4^ and dust transport events^5^, it is an ideal location for understanding the effects of imposed changes on anthropogenic emissions due to the COVID-19 pandemic. Preliminary findings in other urban areas^6^ suggest that lower levels were observed^7^ due to the lockdown measures. Secondary aerosol formation and vehicular traffic (exhaust and non-exhaust emissions) are the main sources affecting concentration levels in Athens^8^, while biomass burning contributes significantly to the aerosol concentrations observed during the cold season^9^. Secondary aerosols are mostly related to sulphate and organics and represent most of the fine particle mass^10,11^.

## Methods and instrumentation

**Figure 1:**
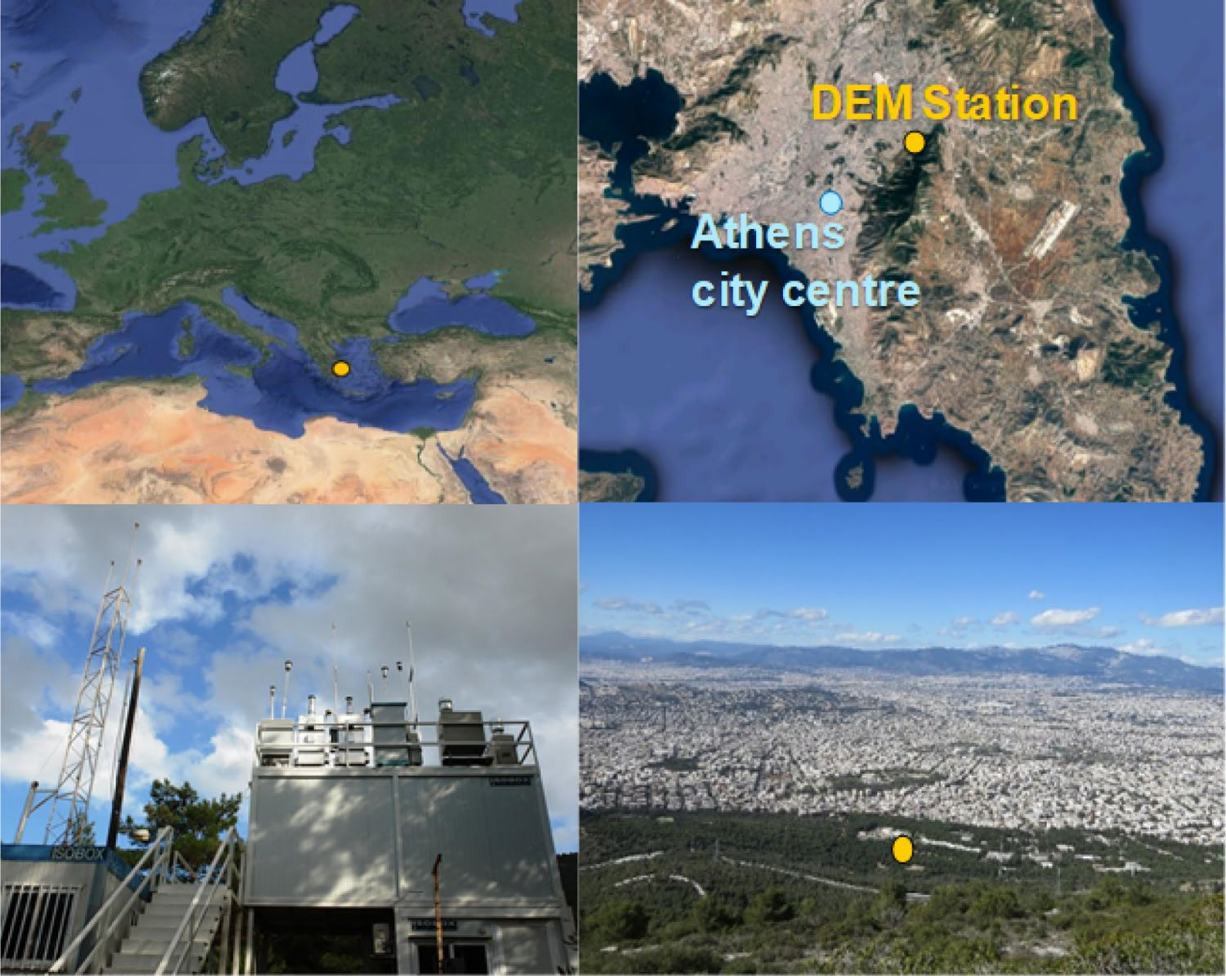
The Demokritos Atmospheric Aerosol Measurement station in Ag. Paraskevi, Athens, Greece, DEM (GAW, ACTRIS). The maps were obtained from Google Maps (maps.google.com) Imagery 2021 Terrametrics, Mapdata 2021 and modified by the authors.

Measurements are continuously conducted at the Demokritos station (DEM station), member of GAW and part of the ACTRIS and PANACEA infrastructures^12^ (37.995° N23.816° E, at 270 m above sea level (asl)). The station is located within the National Centre for Scientific Research “Demokritos” campus, a vegetated area at the foot of Mount Hymettus, about 8 km to the North east from Athens city centre (Fig. 1). It is an urban background station, representative of the atmospheric aerosol in the suburbs of the Athens Metropolitan Area. During the day the station is exposed to advected pollution from the greater area due to mixing within the Athens valley subject to dominant meteorological conditions. An increase in particle number concentration during the night is observed, often in the absence of aerosol particle sources, due to the lowering of the nocturnal boundary layer height (NBLH)^13,14^, while occasional katabatic wind from Mount Hymettus also has an influence^15^.

Measurements presented here refer to the fine aerosol considered as particles with sizes below 2.5 μm, for the following parameters: (i) The aerosol absorption coefficients and equivalent black carbon concentration (eBC) by means of an AE33 dual spot, seven wavelengths (370, 470, 520, 590, 660, 880, 950 nm) aethalometer. The aerosol absorption coefficient was acquired using a multiple scattering correction factor (C_0_) equal to 3.3 in order to correct for multiple scattering by the filter fibers and the scattering of the aerosols embedded in the filter^9^; (ii) The particle number size distribution of atmospheric aerosol in the size range from 10 to 550 nm (electrical mobility diameter), obtained every 5 min by a Scanning Mobility Particle Sizer (SMPS) including a TSI Model 3080L electrostatic classifier (TSI Inc., USA) and a Condensation particle counter (3772 CPC, TSI Inc., USA); (iii) The particle number size distribution for the sizes ranging from 250 nm to 2.5 μm (optical diameter) by an Optical Particle Counter (OPC) (Grimm 107@660 nm laser light wavelength)^16^; (iv) Near-real time elemental (EC) and organic carbon (OC) concentrations in PM2.5 on a 3-h basis, using a Semi-Continuous OC-EC Field Analyzer (Model-4, Sunset Laboratory, Inc., USA), equipped with an in-line parallel carbon denuder and employing the EUSAAR 2 protocol^17^; (v) The chemical composition of non-refractory submicron (PM1) aerosols (i.e. organics, SO_4_^2−^, NO_3_^−^, NH_4_^+^, Cl^−^), with a time resolution of 10 min, by means of a Time-of-Flight Aerosol Chemical Speciation Monitor (ToF-ACSM, Aerodyne Research Inc.)^18,19^; (vi) Atmospheric aerosol PM_2.5_ filter samples were also collected for gravimetric analysis according to EN12341. Meteorological parameters are obtained from standard sensors on a 10 m mast, including Temperature (T), Relative Humidity (RH), Total solar radiation, Rain. As an indication of urban anthropogenic activity and emissions from vehicular traffic the hourly monitored traffic volume in terms of Light and Heavy Duty vehicles (LDV, HDV) was obtained from 3 representative junction points at the Attica Major Ring Road (Attiki Odos) characteristic of traffic volumes at the North AMA sector, where DEM station is situated. The average daily variability for all periods of the study is shown in Fig. 4 in the supplement. All above instruments operate under GAW guidelines at RH nominally below 40% according to GAW specifications, achieved through the use of Nafion dryers and are regularly quality assured in inter-comparison workshops and exercises at the World Calibration Centre for Aerosol Physical Properties (WCCAP)^20^ or the ACTRIS Aerosol Chemical Monitor Calibration Center (ACMCC).

### Methodology for data treatment

#### New particle formation events

In order to identify New Particle Formation events, we used a variant of the procedure described by Dal Maso et al. (2005)^21^. The requirements for a day to qualify as a NPF event day were: (1) a significant increase in the number concentration of particles in the size bins from 10 to 20 nm during the time window 09:00–18:00 Local Time, (2) the size fraction from 10 to 30 nm electrical mobility diameter (N_10-30_) being significantly elevated above the nocturnal background, (3) the burst having a minimum duration of 1 h, and (4) a decrease in N_10-30_ towards the end of the day. The observation of a gradual increase in nucleation mode diameter was not a necessary criterion to qualify a day as an NPF day, but was nevertheless visible in most cases. Nucleation Events have been excluded because of their nature as a new particle formation mechanism in the atmosphere generating particles, which quickly grow in the lowest range (10–30 nm) and show a dramatic increase in terms of number concentrations but are not primary emissions from anthropogenic pollution sources. They cannot be easily distinguished from direct emissions of fresh particles from traffic exhaust^22^ the main source of anthropogenic aerosol in this size range. Only 69 h of time, all in the 2nd lockdown period, were classified as Nucleation Events and were excluded from the dataset used in the current work. It should be noted that if we remove from the dataset the periods of rain, the average particle number concentrations in the different size ranges do not change significantly or the relative change remains the same.

#### Estimation of BC from wood burning and fossil fuel combustion

The absorption coefficients derived from the aethalometer were used in order to estimate the contribution of wood burning and fossil fuel to the total black carbon concentrations, through the application of the Aethalometer model^23^

1 $${b}_{abs}{({\lambda }_{UV})}_{wb}=\frac{1}{1-{(\frac{{\lambda }_{UV}}{{\lambda }_{IR}})}^{{-a}_{ff}}\cdot {(\frac{{\lambda }_{UV}}{{\lambda }_{IR}})}^{{a}_{wb}}}\cdot [{b}_{abs}\left({\lambda }_{UV}\right)-{\left(\frac{{\lambda }_{UV}}{{\lambda }_{IR}}\right)}^{{-a}_{ff}}\cdot {b}_{abs}\left({\lambda }_{IR}\right)]$$

2 $${b}_{abs}{({\lambda }_{IR})}_{wb}={(\frac{{\lambda }_{UV}}{{\lambda }_{IR}})}^{{a}_{wb}}\cdot {b}_{abs}{({\lambda }_{UV})}_{wb}$$

3 $${b}_{abs}{({\lambda }_{UV})}_{ff}={b}_{abs}({\lambda }_{UV})-{b}_{abs}{({\lambda }_{UV})}_{wb}$$

4 $${b}_{abs}{({\lambda }_{IR})}_{ff}={b}_{abs}({\lambda }_{IR})-{b}_{abs}{({\lambda }_{IR})}_{wb}$$

where α_ff_ and α_wb_ are the absorption Ångström exponents for pure fossil fuel combustion and pure wood burning aerosol, respectively; b_abs_(λUV) and b_abs_(λIR) are the absorption coefficients measured at the UV (470 nm) and IR (950 nm) wavelengths, respectively, with the blue (470 nm) channel found to perform better than the traditionally formulated UV channel (370 nm) in the aethalometer model^9^. b_abs_(λUV)_wb_ & b_abs_(λIR)_wb_ and b_abs_(λUV)_ff_ & b_abs_(λIR)_ff_ are the corresponding absorption coefficients at these two wavelengths that are related to wood burning (wb) and fossil fuel combustion (ff). According to the earlier sensitivity study for our area^23^ α_ff_ and α_wb_ values was found equal to 1 and 2 respectively.

### Estimation of aerosol organic mass (OM) from organic carbon (OC) measurements

OM was estimated by multiplying the OC concentrations by a factor representative of the mean molecular to carbon weight ratio, in order to account for the non-carbon atoms of the organic compounds. This factor was set equal to 1.7, according to the urban background character of DEM station as suggested by Turpin and Lim (2001) and references therein^24^.

The diurnal variability of OM was obtained through the mean hourly concentrations measured by the ToF-ACSM PM1 fraction after adjusting the organics' mass based on the measured OC concentrations, in order to account for the total organics, found in PM2.5.

#### Air mass origin

**Figure 2:**
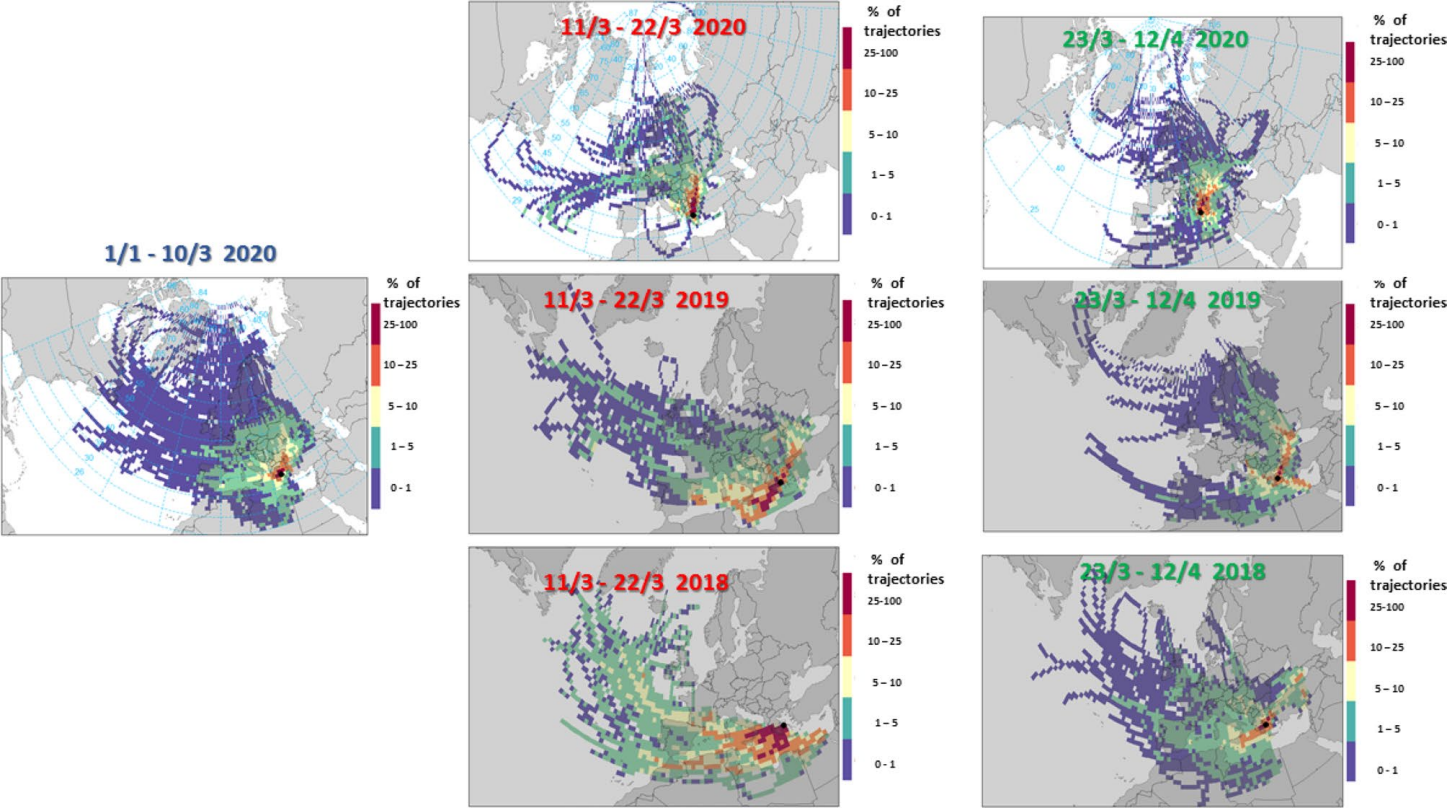
Frequency of spatial origin for air masses arriving at Athens from the greater Region during the proposed reference period (1/1–10/3, 2020) the two lockdown periods under consideration (11/3–22/3, 2020 & 23/3–12/4, 2020) and the respective periods in 2018 and 2019 (Created by R version 4.0.2^27^ and Openair version 2.7–7^28^.

Air mass backward trajectories were calculated for the DEM station using the Hydrid Single-Particle Lagrangian Integrated Trajectory model (HYSPLIT) to gather information about the origin of the observed aerosols and the synoptic patterns corresponding to the period under study^25^. The three dimensional trajectories were computed for the arrival heights of 500 and 1000 m AGL for 120 h backward over Athens, Greece. The methodology for creating the graphical display of air mass frequency in Fig. 2 is given in the supplement.

#### Fuchs surface area

Fuchs surface area is calculated as^26^:

5 $$S_{f} (dm)_{i} = \frac{{12~\pi ^{2} }}{{8.39\xi }}~\frac{{\lambda _{g} d_{{m,i}} }}{{C(Kn_{{m,i}} )}}N(dm)_{i}$$

where $\xi$ is the dimensionless momentum scattering coefficient, ${\lambda }_{g}$ the gas mean free path, ${Kn}_{m,i}$ the particle Knudsen number, $C({Kn}_{m,i)}$ the corresponding Cunningham slip correction factor and ${N(dm)}_{i}$ the number concentration in each size bin (i) as obtained by means of the SMPS. Then, the total Fuchs surface area is calculated as:

6 $$S_{{f,total}} = ~\sum\limits_{i} {N(dm)_{i} } S_{f} (dm)_{i}$$

### Levels and variability of aerosol metrics in the lockdown periods

The periods of lockdown, as described earlier, are separated in 1st Period 11/3- 22/3 2020 and 2nd Period 23/3–12/4 2020 compared to a reference period 1/1–10/3 2020, the latter including all available data from the beginning of the year. The change in air quality metrics is observed as the ratio of the 1st & 2nd period values with respect to the reference values. With negative sign, we observe a reduction and positive indicates an increase. Some initial evaluation on how representative this comparison is with respect to the general variability of urban emissions, weather patterns and other factors affecting aerosol levels, their gaseous precursors and sinks, is also important to present here. The choice of the reference period, against which changes are evaluated is also an important issue. Air pollutants are affected significantly by variability in emissions and meteorology, which affects in turn atmospheric chemistry and formation of secondary species as well as removal mechanisms. We looked carefully at the meteorological data of the respective periods. A seasonal pattern in emissions of carbonaceous aerosol has been documented in Athens, with differences observed between winter and summer^23^. The period of lockdown, which eventually extended for the whole period of April, falls in a transition period of spring when temperature is rising but also is variable from year to year. For example, during the period of lockdown we chose to investigate (11/3–12/4), the temperature remained within comparable levels to winter, as March and early April were considerably cold. The average temperatures during the two-lockdown periods were at 11.5 and 12.2, barely 2 °C on average higher than in winter, while the evening temperatures in all three periods remained the same. This makes these periods to a great extent equivalent in terms of emissions from residential heating including biomass burning. In contrast if one considers the exact same calendar days in previous years as a reference period for the atmospheric conditions and concentration levels of air pollutant species the variability may be high. For example, looking at the weather conditions during the previous 2 years, temperatures in 2018 were at 14.7 °C, a level where use of residential heating is reduced greatly, while in 2019 were again similar to those in 2020. The local meteorology regarding wind directions around our measurement site was considered similar in all periods regardless of the reference period chosen (see supplementary section for wind rose Figure S1). The long-range air mass transport, which may play a role in affecting the background incoming levels of pollutants was examined for both choices of reference periods. In Fig. 2 we observe that air masses during the winter of 2020 were arriving at Athens from the same Northwest to Northeast sectors as observed during the 1st and 2nd lockdown periods, while in the two previous years a dominant sector from west—southwest and even southeast sector was present. These transport patterns indicate that, overall, the periods of the past years are not suitable as reference periods in this study. Looking at the concentration levels observed for some of the representative species characterizing air quality in the urban environment, namely Ozone, NO_2_, fine particle number concentrations and aerosol organic carbon concentration, a mixed picture is emerging (supplement Fig S2). The levels at the previous years do not appear to display a coherent pattern to the extent that their choice as a reference would appear more credible. In general, the variability in concentration levels for several key aerosol and gaseous air quality between 2018 and 2019 is often similar or higher to the differences we want to explain for the observed concentrations in 2020 during the reference and lockdown periods. The effects of changes in anthropogenic activity due to COVID-19 lockdown are evaluated by studying the variability and concentration levels of certain microphysical and chemical parameters listed in Table 1. The complete time series of the air quality concentrations in terms of the aerosol microphysical properties and chemical components, PM2.5 and key gaseous species are displayed in the supplement section (Figure S3).

**Table 1:** Overview of Aerosol Microphysical and chemical parameters measured during the reference, 1st and 2nd lockdown periods as well as auxiliary parameters regarding atmospheric conditions and indicative traffic volumes.

	Metric	Mean values of observations			Change (%) with respect to reference period	
		Reference Period 1/1–10/3	1st lockdown period 11–22/3	2nd lockdown period 23/3–12/4	1st lockdown period (%)	2nd lockdown period (%)
*Aerosol Microphysical Properties*	N_0.01–0.55_ (p/cc)	5969	4703	4165	− 21	− 30
	N_0.01–0.03_ (p/cc)	2182	1071	1161	− 51	− 47
	N_0.03–0.05_ (p/cc)	1474	1036	937	− 30	− 36
	N_0.05–0.10_ (p/cc)	1528	1495	1181	− 2	− 23
	N_0.10–0.55_ (p/cc)	784	1101	886	40	13
	d_g_volume_ (μm)	0.22	0.22	0.23	0	4
	V_0.01–0.1_ (× 10^–6^ cm^3^ m^−3^)	1.13	1.10	0.88	− 2	− 22
	V_0.1–0.5_ (× 10^–6^ cm^3^ m^−3^)	6.41	9.14	8.02	43	25
	V_0.5 -1_ (× 10^–6^ cm^3^ m^−3^)	0.35	0.26	0.29	− 26	− 17
	V_1-2.5_ (× 10^–6^ cm^3^ m^−3^)	0.75	0.36	0.68	− 52	− 9
*Chemical components*	OM* (OC) (μg/m^3^)	2.79	2.94	2.26	6	− 19
	OC/EC	5.24	4.26	4.36	− 19	− 17
	EC (μg/m^3^)	0.56	0.71	0.54	25	− -3
	eBC_wb_ (μg/m^3^)	0.19	0.21	0.18	11	− 5
	eBC_ff_ (μg/m^3^)	0.42	0.5	0.41	19	− 2
	SO_4_^2−^ (μg/m^3^)	1.54	1.66	1.21	8	− 21
	NO_3_^−^ (μg/m^3^)	0.49	0.44	0.23	− 10	− 53
	NH_4_^+^ (μg/m^3^)	0.47	0.54	0.38	15	− 19
	Cl^−^ (μg/m^3^)	0.05	0.03	0.02	− 40	− 60
	NO_2_ (μg/m^3^)	16.0	11.5	7.0	− 28	− 56
	O_3_ (μg/m^3^)	67.6	80.4	76.0	19	12
*Mass* *Conc*	PM_2.5_ (μg/m^3^)	9.10	9.62	10.25	6	13
*Conditions/ Emissions*	Tair (^o^C)	9.5	12.2	11.6	28	22
	Solar Rad (W/M^2^)	108.6	191.1	151.4	76	39
	Hrs of Rain (%)	7.5	2.1	17.1	− 72	128
	Avg Traffic (LDV/hour)	1574	1095	466	− 30	− 70
	Avg Traffic (HDV/hour)	84	68	49	− 19	− 42

A major source contributing to anthropogenic emissions in Athens is vehicular traffic. A measure of the traffic intensity around the city can be drawn from the Average daily counts for Light Duty Vehicles (LDV) and Heavy Duty Vehicles (HDV). This was derived from hourly data of the Attiki Odos Ring road at three major junctions of the central section of the Motorway running across the North part of the Athens Metropolitan area (can be found in the supplement Fig. S4). We find in Table 1 that during the 2nd phase a very significant for LDVs and considerable for HDVs reduction in volumes is observed, while in the 1st period a considerable drop for LDVs and only a small drop for HDVs numbers is visible.

The microphysical metrics regarding particle number and volume display a complex behaviour. In general, total particle number is reduced by 20 and 33% in the 1st and 2nd periods respectively. However, this change is not uniform across the particle size range. The nuclei mode, governed by primary emissions mainly from the local urban traffic sources^29–31^, is most significantly reduced to half the concentration observed in the reference period. The larger Aitken and accumulation modes show progressively smaller reductions, while the accumulation mode during the 1st lockdown period shows an increase by 29%. We often find in Athens for the accumulation mode aerosol to be characteristic of regional pollution or episodic transport^4^. We also show in Fig. 2 that the air mass origin appeared quite uniform during all study periods. A slight persistence of air masses originating from North Eastern Europe during both lockdown periods with respect to the reference period, cannot explain the difference in the level of increase in some microphysical parameters in Table 1 observed during the 1st lockdown period with respect to the decrease in the 2nd lockdown period. This first finding will be examined further in terms of the dynamics of primary emissions and gaseous precursors affecting in turn the growth of all aerosol size classes. The same pattern somewhat intensified is visible in the carbonaceous and other chemical components. Despite the decline in numbers, we also observe that particle diameter, as an average geometric mean, remains stable, so the aerosol volume and mass do not change in the same way as particle numbers (Table 1). Further information about specific pollutants we obtain looking at other major aerosol parameters i.e. OC, EC. We observe that these components, which are known to have several sources, follow a different trend compared to ultrafine aerosol and show an increase during the 1st lockdown period. Almost in all metrics related to the carbonaceous aerosol we observe an increase during the 1st lockdown period. Despite the reduction in the number of cars (LDV in Table 1), the lower OC/EC ratio may originate from increased relative share of HDV (diesel) traffic, which is not reduced as much as the LDVs, as a fraction of total traffic during this period. During the 2nd lockdown period, no significant decrease in EC concentration levels during daytime is found, but OC is lower during all times, in comparison to the 1st period. Despite the uncertainty introduced by some of the features displayed by the carbonaceous aerosol, additional information on the origin and changes caused by the lockdown measures are revealed by valuable data for major chemical components (SO_4_^2−^, NO_3_^−^ and NH_4_^+^)_,_ measured at an hourly time resolution by a ToF-ACSM system. Results from Table 1 clearly indicate that the increase in the 1st lockdown period experienced in the carbonaceous aerosol and the accumulation mode of particles in terms of number and volume is again confirmed by SO_4_^2−^, NH_4_^+^ and sum of organic matter (OM). NO_3_^−^ is the only component showing a decline at the 1st lockdown period, while all components decline between 20 and 30% and Nitrate more than 50% at the 2nd period. These findings indicate significant impacts from the reduced anthropogenic activity on specific aerosol metrics, i.e. the ultrafine particle number and secondary aerosol nitrates, which are both partly linked to the traffic emissions of gasoline LDVs. On the other hand, the PM_2.5_ mass concentrations remain unaffected by the overall changes and display a small increase during the two lockdown periods. This is to some extent observed and in the sum of the aerosol volume fractions obtained by the integrated averages of the real time instruments. PM2.5 in Athens is often not exactly representative of the fine aerosol mass and contains a tail end of the coarse fraction^32^.

### Time resolved analysis over the 24 h daily cycle

**Figure 3:**
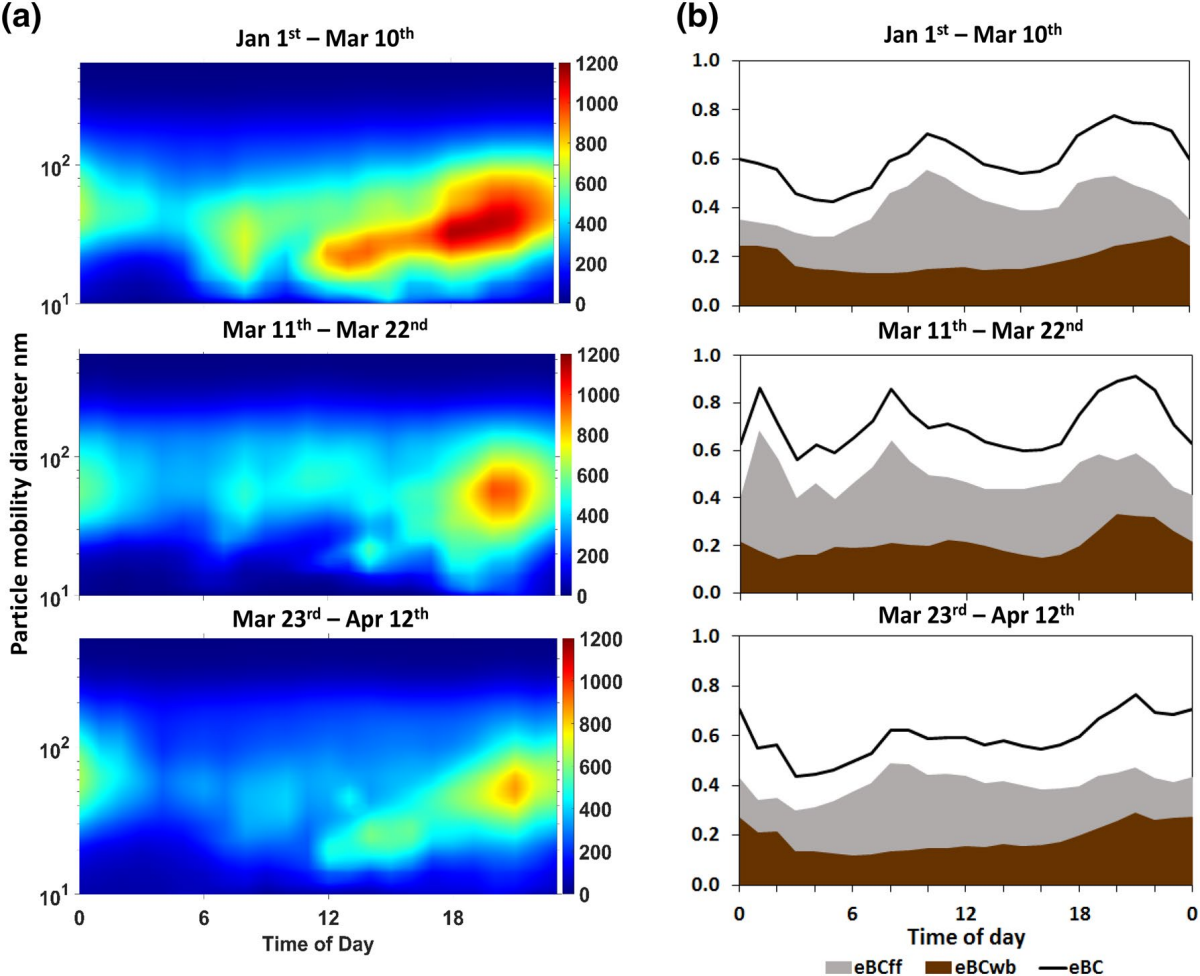
Mean observed variability of hourly concentrations for (**a**) the Aerosol Number Size Distribution and (**b**) the Black Carbon Concentration resolved for the fossil fuel (eBC_ff_), wood burning (eBC_wb_) components and their sum (Total eBC) in the Athens urban background aerosol during the Reference and the 1st and 2nd lockdown periods.

A further elucidation of this change in the observed statistics and the dynamics active during these periods is possible by plotting in Fig. 3(a) the average 24 h number concentrations of the detailed size distribution in terms of a colour map of the hourly values. Considering the background location of our measurement site, we observe that during the reference period the morning traffic peak is well visible, while it gradually diminishes in the 1st lockdown period and finally disappears in the 2nd period. The higher aerosol load normally arrives later during the course of the day, when conditions favour mixing and dispersion of the generated aerosol across the Athens valley. During the lockdown periods, the nuclei and Aitken modes continue to appear at much lower concentrations with a variable intensity which maybe also affected by transport and dispersion mechanisms as well as exchange with regional air masses. During the evening hours, concentrations peak due to additional emissions by residential heating and a developing local inversion/nocturnal boundary layer^9,31^. The potential effect of a systematic change in the height of the boundary layer in the concentration levels was investigated. The boundary layer height (BLH) dataset was obtained from the ERA-Interim global atmospheric reanalysis^33,34^ produced by European Centre for Medium-Range Weather Forecasts (ECMWF). Forecasts were initiated at 00:00 and 12:00 UTC, and provided the 3-hourly (time step) boundary layer heights with a spatial resolution of 0.125° × 0.125°. It was found that there was an average decrease of 25.3% during the 1st lockdown period and 7.2% during the 2nd lockdown period in comparison to the reference period (Figure S5 in the supplement).

**Figure 4:**
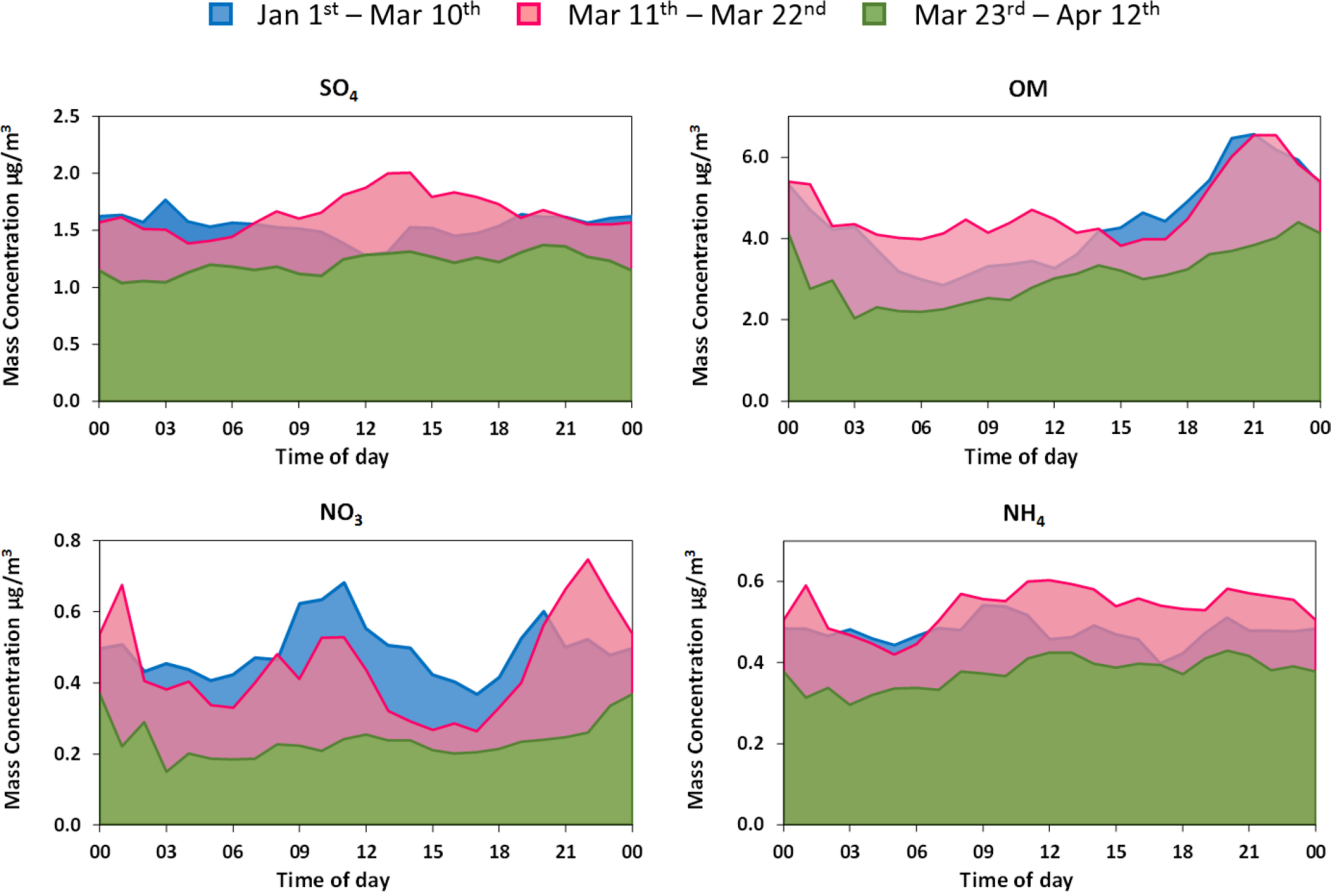
Mean observed variability of hourly concentrations for major Organic (OM) and Inorganic (SO_4_^2−^, NO_3_^−^ & NH_4_^+^) components in the Athens urban background aerosol during the Reference and the 1st and 2nd lockdown periods.

This indicates that the reference and 2nd lockdown periods are equivalent in terms of the BLH conditions, while the 1st period was characterized by relative lower mixing layer heights (i.e. increased atmospheric stability) which might explain qualitatively the pronounced increase of some parameters with no or little decline and the relatively weaker decrease of the traffic related species (Supplementary Figure S7). Generally, much higher concentrations are observed in the reference period with a gradual significant decline in the 2 lockdown periods. Taking into account that temperatures are very similar along all periods, residential heating emissions are expected to be continuously active and in fact are probably increased since people are staying indoors and make use of the residential heating on a continuous basis. The observed decline in ultrafine particles as a total (< 100 nm) is then only due to the decline of traffic emissions. Both traffic and residential heating contribute to the carbonaceous aerosol, so changes there are visible only after detailed examination of the 24 h cycle. Looking at the normalized OC/EC ratio, we find it lower in the early morning hours indicating the persistence of diesel emissions which arises from the smaller decline of HDVs due to services and supply chain operations. The OC/EC ratio most clearly shows an increase in the 2nd lockdown period during the early evening, when the residential heating becomes partly more dominant than traffic (higher OC/EC ratio during 18:00–21:00 (Supplementary Fig. 4). Regarding the origin of carbonaceous aerosol from the two major general categories of fossil fuel (ff) and wood burning (wb) emissions, we get a well-quantified picture for the three periods from the aethalometer model^35^ applied on the equivalent BC (eBC) data, displayed in Fig. 3b in terms of eBCff and eBCwb. Here we normally (reference period) observe the expected rise of the WB component during the evening hours and a flat background during the rest of the day when residential heating is not fully active. However, there is an observable rise in the 2nd lockdown period around the middle of the day due to the full day use of the residential heating and this appears to coincide with the midday increase observed in the normalized OC/EC ratio (Supplementary Figure S6). However, we observe that the calculated secondary organics^36^, sulphates and nitrates all display a varying change from decline to increase, indicating that there are more factors influencing the aging of particles. Heterogeneous chemistry and equilibrium with condensable species are usually the factors driving the overall process in the fine aerosol^37,38^. We draw further evidence to the above features observed during the lockdown periods, when looking into the average daily variability of major inorganic aerosol species and the Organic aerosol matter (Fig. 4).

SO_4_^2−^ and NH_4_^+^ are not generally affected by the daily dynamics because they are part of the homogeneous aerosol background, with the exception of small details in the NH_4_^+^, which in a small part is associated with NO_3_^−^. The latter is strongly modulated according to the 24 h urban activity cycle just like the ultrafine particles. It is observed that the reductions in traffic activity during the 2nd lockdown period had a strong effect, eliminating the fingerprint of the 24 h cycle in Nitrate formation above the local or regional background levels. It is concluded that secondary NO_3_^−^ evolving from NOx emissions in the city and ultrafine nuclei have their origin in traffic and this source is governing the dynamics and lifetime of these air quality metrics. It is known that SO_2_ emissions have declined very substantially in AMA for the last decades^39^. Ammonium sulphate is a pollutant known to originate mostly from outside the urban area and beyond^40^ and it is therefore related to the incoming aged aerosol, apart from some industrial sources and shipping, which were also affected by reduced economic activity.

Extra information on the dynamics of these changes can be drawn by looking at the modal structure of the ultrafine and accumulation modes. There we observed a significant difference in aerosol aging. First, the calculated aerosol Fuchs surface area^26^ and corresponding condensation sink (CS) is found higher for the 1st lockdown period than the 2nd and lowest for the reference period (Supplementary Fig. 7). The growth rates (GR) calculated^41^ for evolving nuclei to large Aitken modes (from 20 to 70 nm) are found to be markedly different (5.4, 4.5 and 3.7 nm/h for the 1st lockdown period, the 2nd and the reference periods respectively). This has been documented in different environments^42^ where a positive relation between GR and CS was found and a strong contribution of the (semi-condensable) vapours to the build-up of CS was established. Here, we observed that despite the decline of the nuclei and small Aitken mode, the increased growth rate maintains gas to particle conversion regardless the ongoing reduction in gaseous precursors.

The resulting fine aerosol volume concentration of this unimodal distribution corresponds to the calculated growth rate of the smallest fresh modes, previously outlined with the highest growth rate corresponding to the highest volume in the 1st lockdown period, and the lowest growth rate corresponding to the lowest volume during the reference period (Figure S5).

We confirm for the first time in these different urban conditions of anthropogenic activity reduction due to COVID-19, that the relatively larger particles in the ultrafine modes exert the biggest influence in the uptake of condensable vapours^42,43^. The Geometric Mean number diameters of the Aitken and accumulation modes are the highest in the 2nd period of lockdown and this explains the increased aerosol volume in the range between 0.1–0.5 μm.

**Figure 5:**
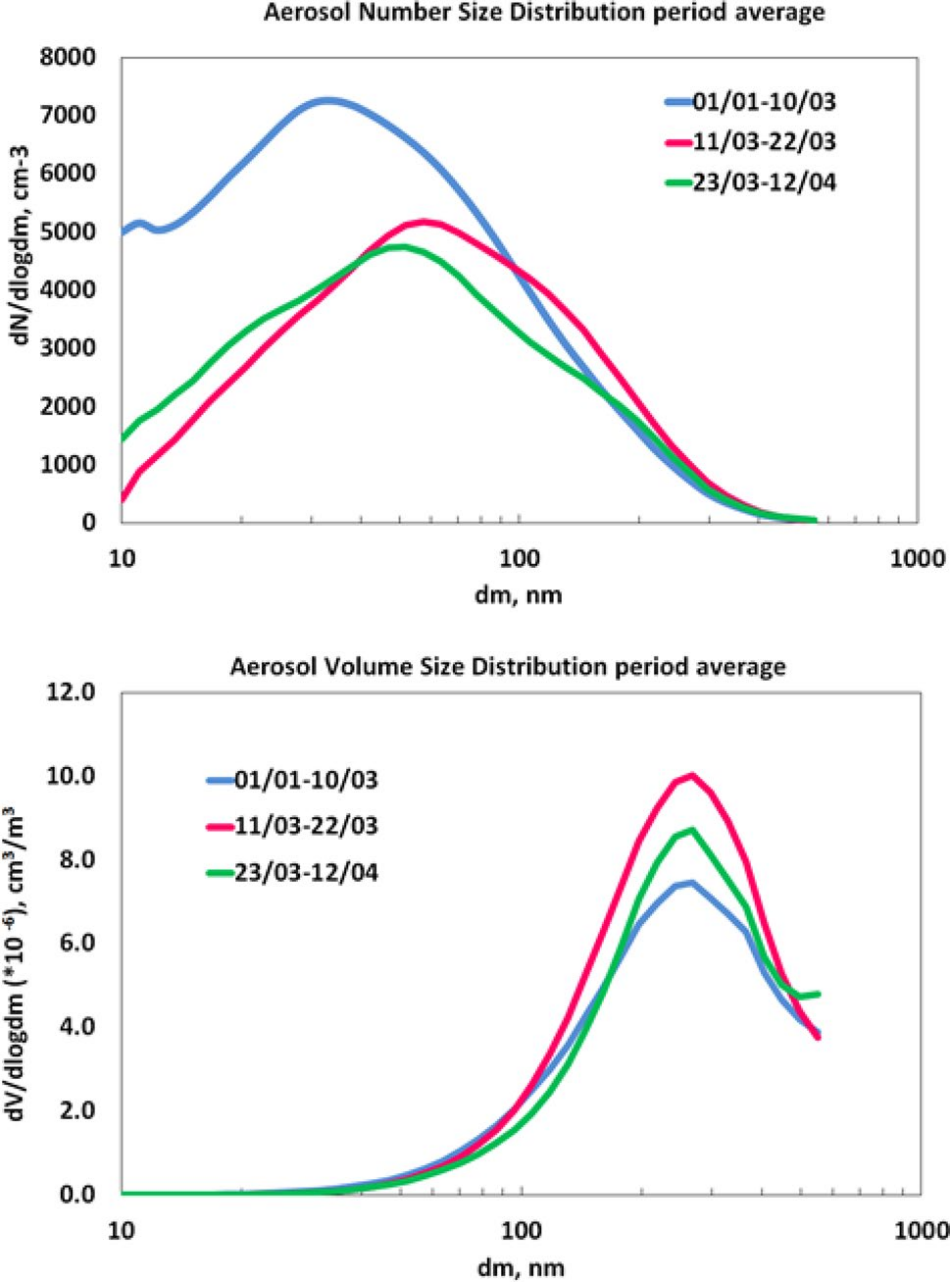
Aerosol Number and volume Size distribution during the lockdown and reference periods.

Here we observed that despite the decline of the nuclei and small Aitken mode, and primary emissions, the increased growth rate maintains gas to particle conversion regardless the ongoing reduction in gaseous precursors. This is also reflected in the overall period average of the aerosol size distribution, if we consider first the number size distribution with the evident decline of the nuclei and Aitken modes and the volume distribution (Fig. 5).

The average GRs showed a positive correlation with both Fuchs surface area, which is directly proportional to condensation sink (i.e. CS describes the loss rate of condensable vapours due to their condensation onto aerosol particles), and O3 concentrations. In addition, the GRs measured in this study are too large to be explained with sulphuric acid as the major condensing vapour^44^. These provide an indication that condensation of semi-volatile organics onto the particle surface area is driving most of the growth. In general, the oxidation reactions of VOCs with ozone, hydroxyl radical and nitrate radical all are capable of producing vapours that contribute to the particle growth^42^. It has been documented in different studies that the particle growth rate is higher in the Aitken mode than in the nucleation mode which follows from the growth caused by partitioning of semi-volatile vapours^42,46^. As the particles get larger, a greater fraction of the semi-volatile vapors can condense onto the particles and cause them to grow. This result is reasonable in light of the thermodynamics of evaporation, as Kelvin effect affects the mass flux onto the smallest particles, decreasing the maximum condensing fraction of the total available condensable material^37,45^. Although several of these studies are focused or investigate the process of nucleation from gaseous precursors to clustering and further particle growth, the process of growth is governed by the same reactive and condensing organic and inorganic agents that lead to growth even when nucleation is not active due the effect of the CS outcompeting the process of new particle formation. As demonstrated in Yan et al. (2021), growth in larger sizes is dominated by Oxidized Organic matter of variable volatility^47^. Although we do not present here measurement of SO_2_ and VOCs, we can observe that the total Organic mass and sulphates show a small (during the 1st period) to a moderate decline (during the 2nd period) in contrast to a much sharper decline in NOx and resulting particulate NO_3_^−^ (Table 1) This coupled with increased O_3_ concentrations fits with the results of increased growth rates observed and slightly increased aerosol volume shown in the supplement (Fig. S7). The sharp reduction in NO2 and parallel increased condensable material on the fine aerosol fraction is also in line with findings for NO_2_ inducing variable growth rates with aerosol size or partly supressing growth as observed in the CLOUD chamber experiments^48^.

Overall, it has been documented in different studies that the particle growth rate is higher in the Aitken mode than in the nucleation mode which follows from the growth caused by partitioning of semi-volatile vapours^36,40^. As the particles get larger, a greater fraction of the semi-volatile vapors can condense onto the particles and cause them to grow. This result is reasonable in light of the thermodynamics of evaporation, as Kelvin effect affects the mass flux onto the smallest particles, decreasing the maximum condensing fraction of the total available condensable material^39,41^.

## Conclusions and implications

It is concluded that the overall impact on key aerosol parameters and air quality metrics observed at the urban background is not a reduction in concentration of pollutants proportional to a general reduction in anthropogenic activity. Fresh traffic emissions and their aerosol products in terms of ultrafine nuclei particles and nitrates showed a significant reduction especially during the 2nd period (40–50%). Carbonaceous aerosol both from fossil fuel emissions and biomass burning, as well as aging ultrafine and accumulation mode particles showed an increase of 10–20% on average, before showing a decline (5 to 30%), with this behaviour governed mostly by residential heating emissions. The impacts of induced changes in anthropogenic emissions are finally governed by the dynamics of aerosol aging in the urban environment. Removal of small nuclei and Aitken modes increased growth rates and migration of condensable species to larger particles maintaining aerosol volumes. PM_2.5_ mass concentration, which is the regulated air quality parameter, did not show any decrease in its levels in the urban background.

## Supplementary Information

Supplementary Information.
