# Author Correction: Aerosol microphysics and chemistry reveal the COVID19 lockdown impact on urban air quality

**DOI:** 10.1038/s41598-021-99947-w

**Published:** 2021-10-08

**Authors:** Konstantinos Eleftheriadis, Maria I. Gini, Evangelia Diapouli, Stergios Vratolis, Vasiliki Vasilatou, Prodromos Fetfatzis, Manousos I. Manousakas

**Affiliations:** 1 Environmental Radioactivity Laboratory, INRASTES, NCSR Demokritos, 15310 Ag. Paraskevi, Athens, Greece; 2 LAC, Paul Scherrer Institute, Villigen PSI, Switzerland

Correction to: *Scientific Reports* 10.1038/s41598-021-93650-6, published online 14 July 2021

The original version of this Article contained an error in Affiliation 1, which was incorrectly given as ‘Environmental Research Laboratory, INRASTES, NCSR Demokritos, 15310 Ag. Paraskevi, Athens, Greece’. The correct affiliation is listed below:

Environmental Radioactivity Laboratory, INRASTES, NCSR Demokritos, 15310 Ag. Paraskevi, Athens, Greece

The original Article has been corrected.

