# Supplementary material for: Aerosol microphysics and chemistry reveal the COVID19 lockdown impact on urban air quality
Source: Sci Rep. 2021 Jul 14;11:14477. doi: 10.1038/s41598-021-93650-6 (PMC8280149; doi:10.1038/s41598-021-93650-6)

Supplement

Aerosol microphysics and chemistry reveal the COVID19 lockdown impact on urban air quality

Konstantinos Eleftheriadis[1*], Maria I. Gini[1], Evangelia Diapouli[1], Stergios Vratolis[1], Vasiliki Vasilatou[1], Prodromos Fetfatzis[1], Manousos I. Manousakas[1,2]

[1]ERL, INRASTES, NCSR Demokritos, 15310 Ag. Paraskevi, Athens, Greece

[2]LAC, Paul Scherrer Institute, Villigen PSI, Switzerland

Supplement on methods

Air mass origin

To identify the spatial origin of the various aerosol components, air mass back-trajectories arriving at the site have been analyzed by statistical methods. For the analysis of the back trajectories the openair package was used[1]. Openair is an R package primarily developed for the analysis of air pollution measurement data but which is also of more general use in the atmospheric sciences. The package consists of many tools for importing and manipulating data, and undertaking a wide range of analyses to enhance understanding of air pollution data.

The trajectories were calculated using the NOAA (National Oceanic and Atmospheric Administration) HYSPLIT 4.0 model (Air Resources Laboratory 2017)[2,3]. The model calculation method is a hybrid between the Lagrangian approach, using a moving frame of reference for the advection and diffusion calculations as the trajectories or air parcels move from their initial location, and the Eulerian methodology, which uses a fixed three-dimensional grid as a frame of reference to compute pollutant air concentrations[3].

In this study, 120-hour (5 days) backward air mass trajectories arriving over the location of the sampling site, were computed. Global data assimilation system (GDAS) meteorological files have been used for backward trajectory computation. The data have a spatial resolution of 1-degree longitude and latitude. Detailed information regarding the GDAS database are provided in Stein et al. (2015) and the NOAA Atmospheric Research Laboratory website. The backward trajectories were computed every three hours (ending at 00:00, 03:00, 06:00, 09:00, 12:00, 15:00, 18:00 and 00:00 UTC hours) for each day of the period of study at 500 m above ground level (agl).

References

1.Carslaw, D. C. and Ropkins, K.: Openair - An r package for air quality data analysis, Environ. Model. Softw., 27–28, 52–61, doi:10.1016/j.envsoft.2011.09.008, 2012.

2. Rolph, G., Stein, A. and Stunder, B.: Real-time Environmental Applications and Display sYstem: READY, Environ. Model. Softw., 95, 210–228, doi:10.1016/j.envsoft.2017.06.025, 2017.

3. Stein, A. F., Draxler, R. R., Rolph, G. D., Stunder, B. J. B., Cohen, M. D. and Ngan, F.: Noaa’s hysplit atmospheric transport and dispersion modeling system, Bull. Am. Meteorol. Soc., 96(12), 2059–2077, doi:10.1175/BAMS-D-14-00110.1, 2015.

Supplementary figures

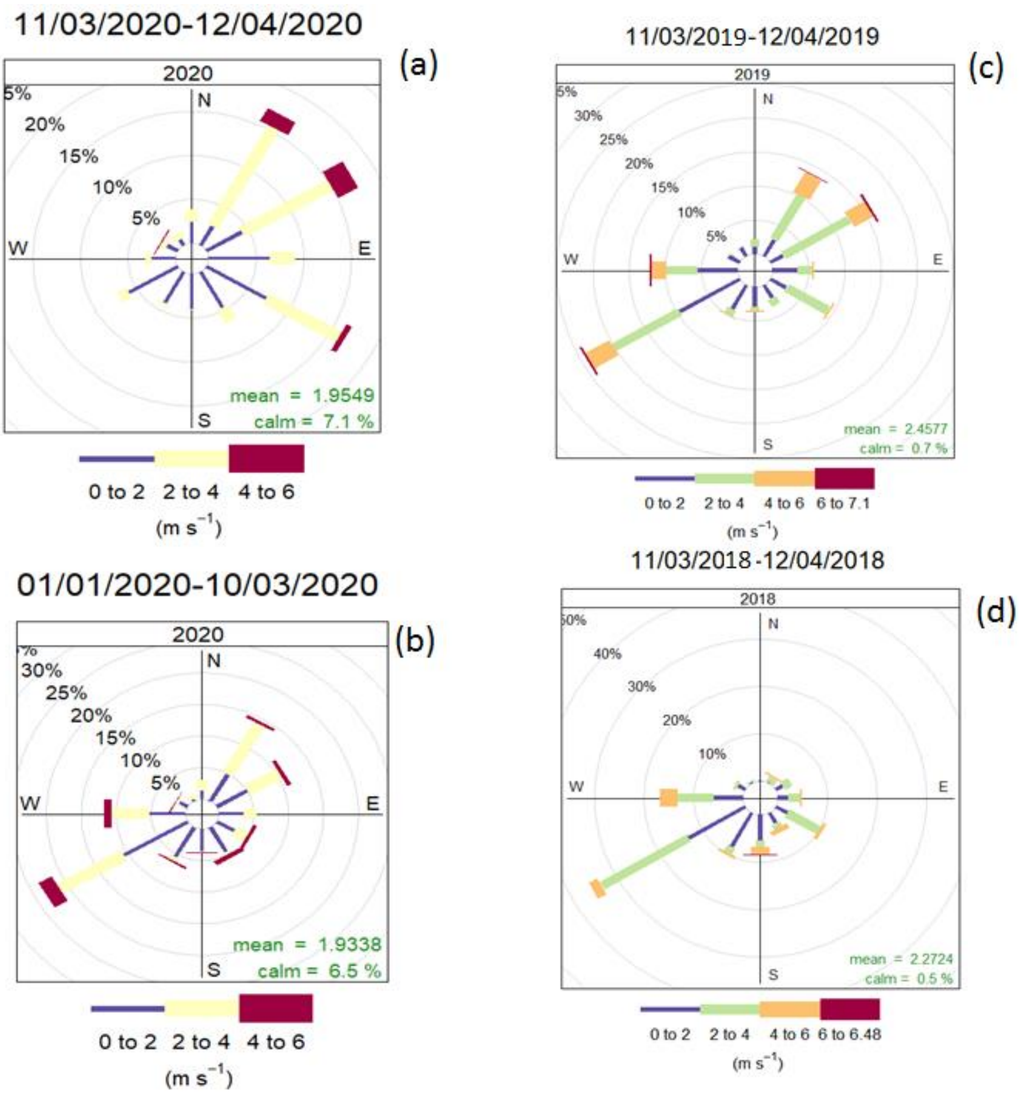

Supplementary Fig. 1 Local wind rose frequencies of occurrence at the Demokritos Atmospheric Aerosol Measurement station in Ag. Paraskevi, Athens, Greece, for the combined lockdown period under consideration (11/3-12/4 2020) (a) and the same period during the proposed reference conditions in 2020 (b) and the past years 2019 and 2018 (c) & (d)

Supplementary Fig. 2: Concentration levels of key air quality species during the proposed reference period (1/1-10/3, 2020) the two lockdown periods under consideration (11/3-22/3 2020 & 23/3-12/4 2020) and the respective periods in 2018 and 2019.

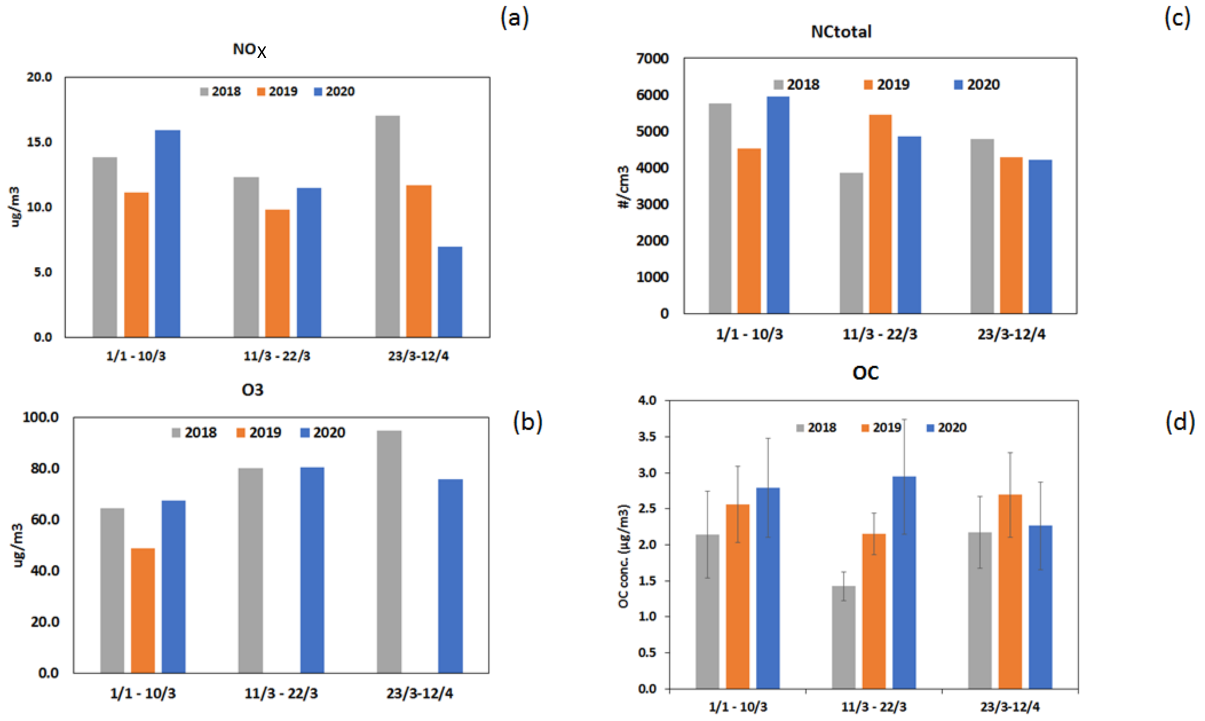

Supplementary Fig. 3: Time series of the air quality concentrations summarized in table 1 in terms of the aerosol microphysical properties and chemical components, $PM_{2.5}$ and key gaseous species for all three periods under study (1/1-10/3 2020, 11/3-22/3 2020 & 23/3-12/4 2020).

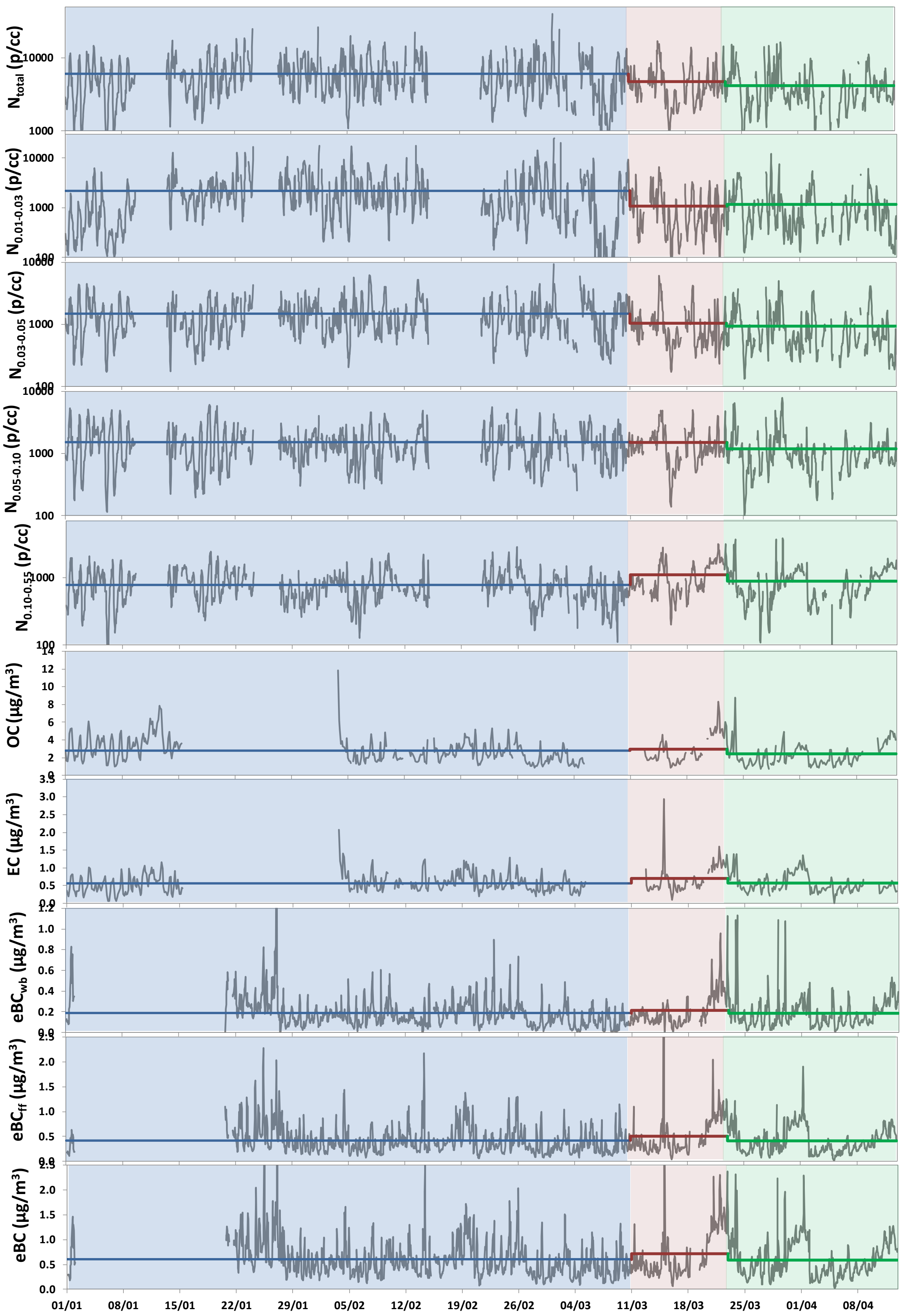

Supplementary Fig. 3 (continued)

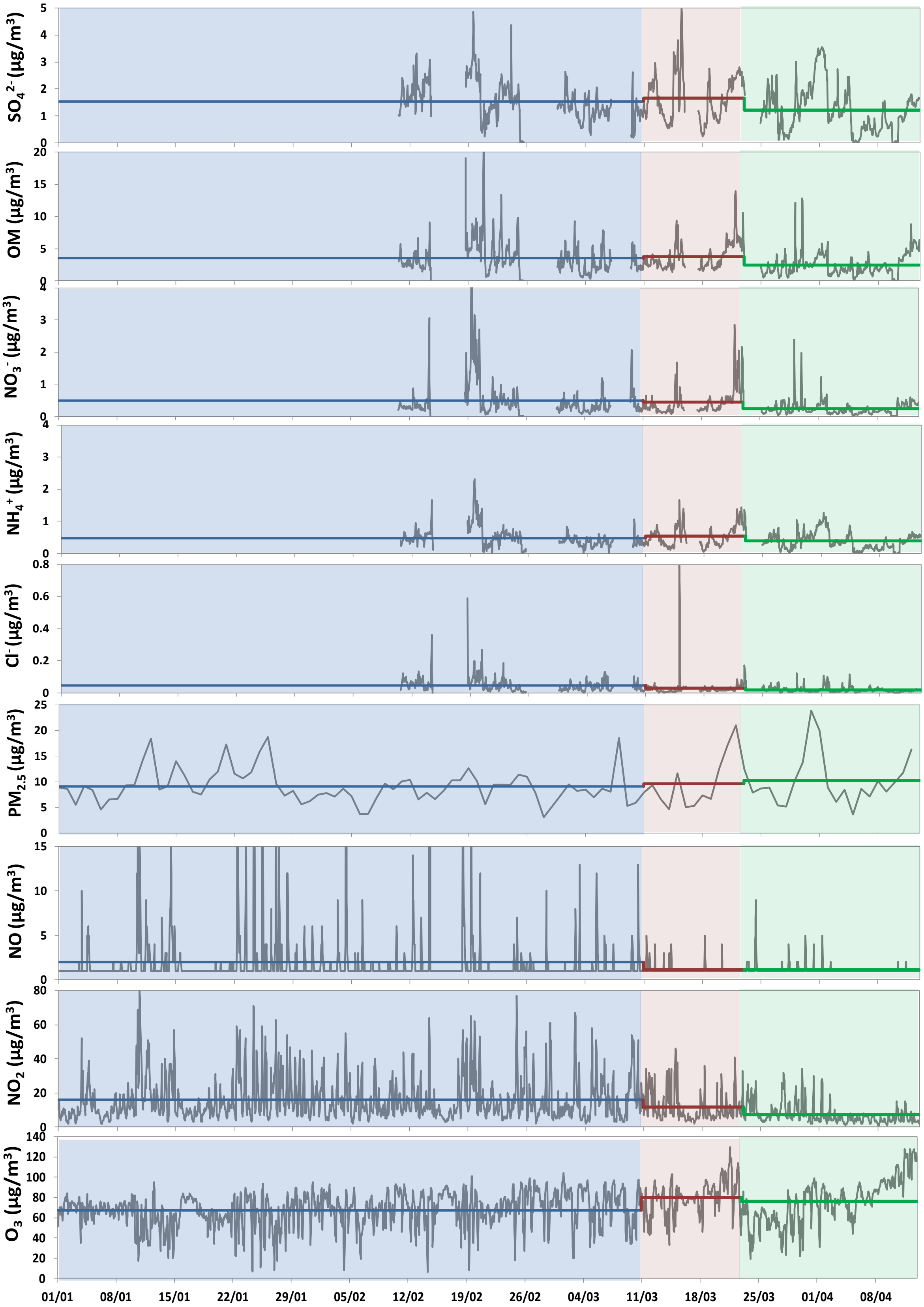

Supplementary Fig. 4: Diurnal variability of the 3-h aerosol OC/EC concentration ratio for each period normalized by the corresponding mean value of the ratio for this period.

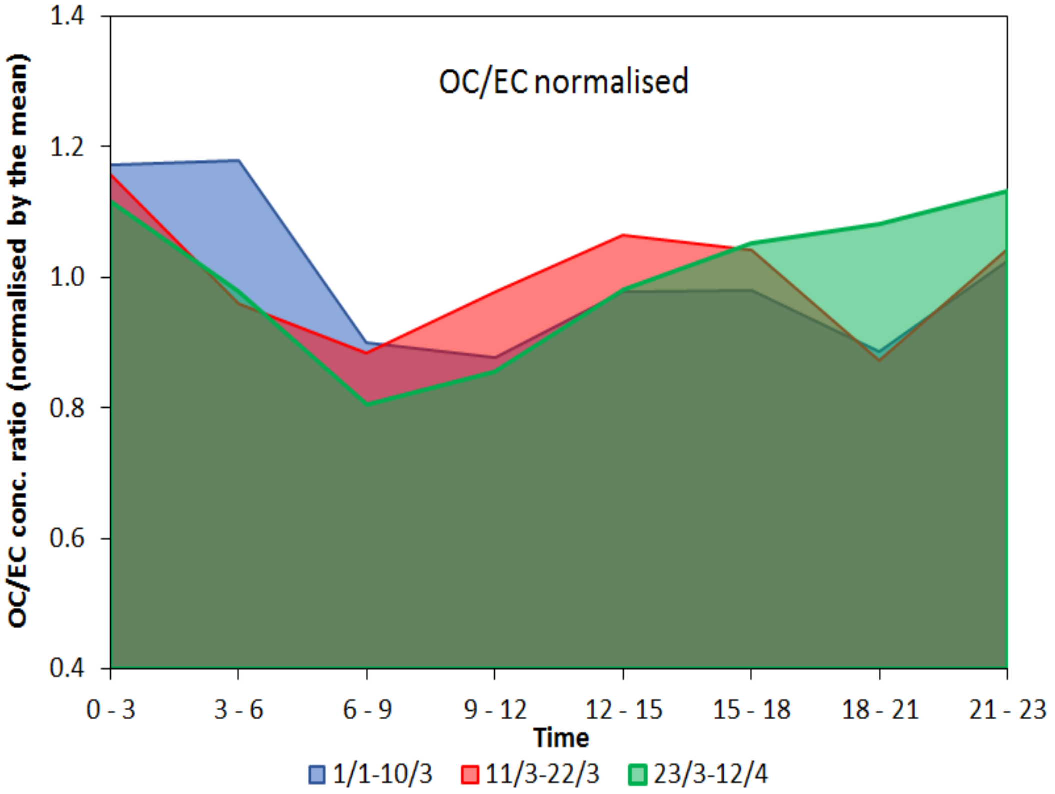

Supplementary Fig. 4: Diurnal variability of the hourly aerosol Fuchs surface area for each period.

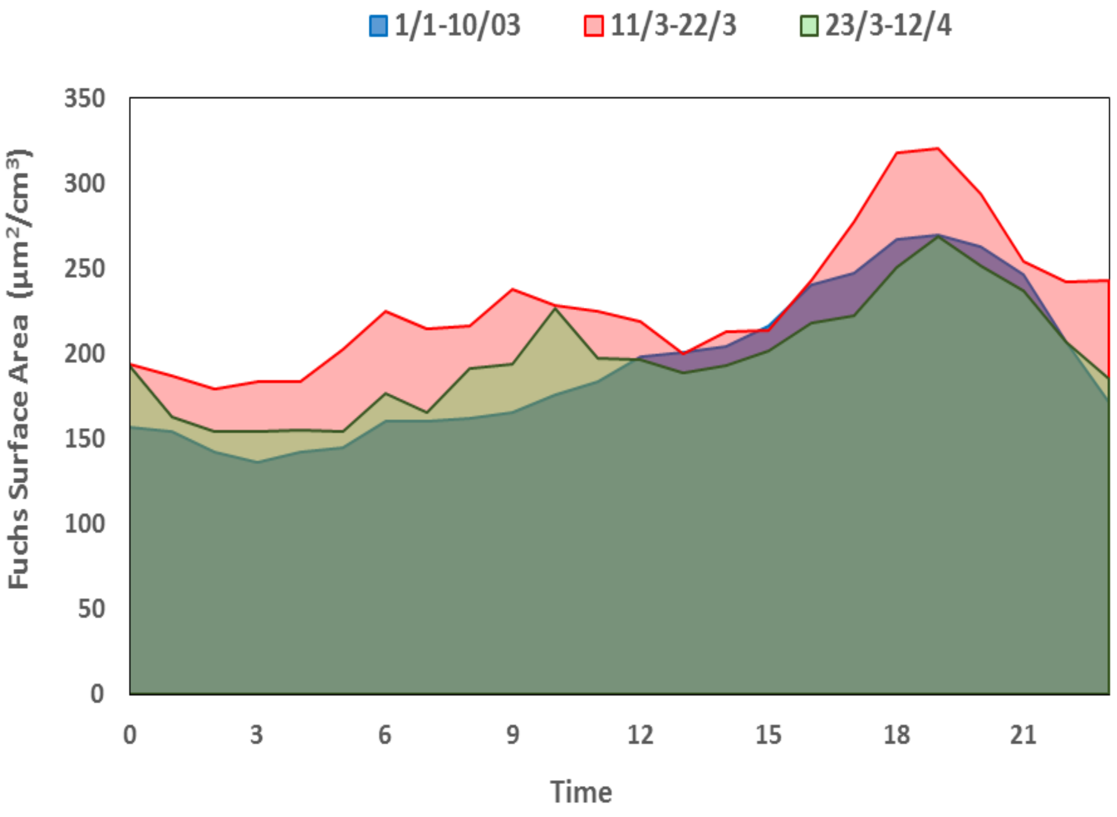

Supplementary fig 5. O_3, Fuchs Surface area (S_f) and NO_2 with respect to average growth rates during the 3 periods under study.

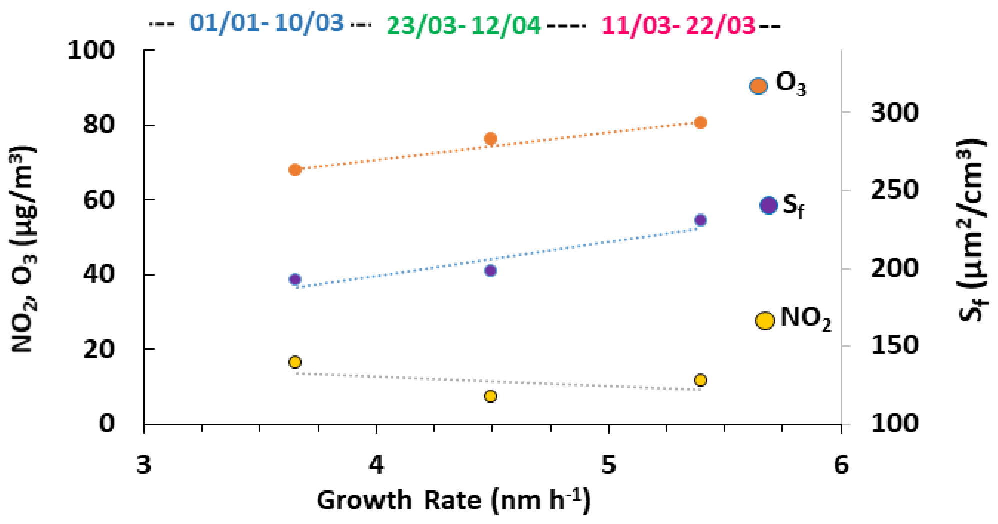

Supplement: Supplementary file 1 — Supplementary Information. [file 41598_2021_93650_MOESM1_ESM.pdf]
